# Effects of Ergomotor Intervention on Improving Occupational Health in Workers with Work-Related Neck-Shoulder Pain

**DOI:** 10.3390/ijerph16245005

**Published:** 2019-12-09

**Authors:** Billy C. L. So, Grace P. Y. Szeto, Rufina W. L. Lau, Jie Dai, Sharon M. H. Tsang

**Affiliations:** 1 Department of Rehabilitation Sciences, The Hong Kong Polytechnic University, Hong Kong 999077, SAR, China; jay.dai@redcross.org.hk (J.D.); sharon.tsang@polyu.edu.hk (S.M.H.T.); 2 School of Medical & Health Sciences, Tung Wah College, Hong Kong 999077, SAR, China; graceszeto@twc.edu.hk (G.P.Y.S.); rufinalau@twc.edu.hk (R.W.L.L.)

**Keywords:** ergonomics, neck pain, motor control, work-related musculoskeletal disorders

## Abstract

(1) Background: Work-related neck and shoulder pain (WRNSP) are common problems, and past occupational research has focused on ergonomic interventions such as adjusting workstations while physiotherapists have traditionally focused on teaching exercises to improve posture and movement control in the clinical setting. The current study aimed to integrate these two approaches and evaluate the immediate and long-term effects of such interventions on occupational exposure outcomes. (2) Methods: A total of 101 patients diagnosed with WRNSP were randomized into 2 groups: Control (CO) group (*n* = 50) and ergomotor (EM) group (*n* = 51). Participants in the control group had 12 weeks of usual care (conventional physiotherapy) while participants in the EM group received an integrated program with tailor-made motor control training and ergonomic advice for 12 weeks. (3) Results: Both groups achieved significant improvement in pain and functional outcomes at post-intervention. The EM group also reported significantly improved scores in terms of perceived exertion in the job-related physical demands (JRPD) and the short form workstyle questionnaires compared to the control group. (4) Conclusions: The results suggest that ergomotor intervention may be more effective in producing favorable occupational health outcomes compared to conventional physiotherapy.

## 1. Introduction

Work-related neck-shoulder pain (WRNSP) are common problems among different occupational groups [1]. These include office workers [2], healthcare workers such as nurses [3] and therapists, engineers, and manual workers (e.g., construction site workers). WRNSP affect millions of working populations all around the world. Because of the advancement of technology and the evolution nature of work, more workers are exposed to prolonged static posture and repetitive upper limb actions [4]. These two factors are widely recognized as the most common occupational risk factors contributing to WRNSP [1,4]. In the United States, the occurrence of WRNSP has been reported at a high rate with around 56–65% of all occupational injuries and its associated direct and indirect annual costs were over $2 billion [5]. In other developed European countries including the Netherlands and Denmark, the prevalence of WRNSP has been reported to be 20–40% [1]. In Hong Kong, there is little epidemiological data available on WRNSP and the Department of Labor has very stringent definition of compensable “occupational diseases” so the reported statistics were very low with less than 400 cases per year [6]. Recent studies in Hong Kong show that the prevalence of WRNSP is high among health care workers [7], construction industry [8], and catering industry [9]. 

Research from the occupational perspective often involve physical ergonomic interventions, organizational interventions, workplace modifications, and engineering controls [4]. From the clinical perspective, these workers with chronic neck-shoulder pain may seek medical advice and treatment from physiotherapists. Conventional treatment often involve therapeutic exercises and manual therapy to relieve joint stiffness or muscle tension. A few systematic reviews have been published to evaluate the effectiveness of different interventions for WRNSP or related disorders, and the results are inconclusive [4,10,11,12]. 

There have been a few recent reports supporting the effects of workplace-based exercise training programs on reducing work-related neck pain. [13,14,15] However, these studies have generally reported the change in self-reported pain scores and there are no measures to indicate how the interventions affect their work habits or occupational exposure factors. It is well-known that both physical and psychosocial factors are at play in contributing to work-related musculoskeletal disorders [16,17,18,19]. There are some validated instruments that can measure these factors and it will be useful to evaluate whether workplace interventions can effectively change the physical and psychosocial exposure in the workers. 

Different ergonomics risk factors may be present in different occupations or job titles. However, most of the workers are not aware of these ergonomics risk factors. The research on providing a universal assessment of physical and psychosocial risk factors for work-related musculoskeletal disorders has not been conclusive. Physiotherapists provide individual assessment from a clinical perspective while job task analysis is also an important aspect to be considered. Integration of ergonomics education, proper working posture and muscle control training will be a good solution for workers with work-related disorders. Ergomotor (EM) approach is an innovative approach by integrating (1) workplace-based ergonomics knowledge transfer, (2) biofeedback motor control facilitation and (3) tailor-made neck and shoulder motor re-education exercises, (4) how to transform all these newly learned knowledge and movement habits into the daily work practice. 

The physical job demands of participants from different job titles were assessed using the job-related physical demand (JRPD) that was first developed by Feuerstein and associates, to evaluate the physical aspects of different workers that included both sedentary type of work such as office workers as well as manual handling tasks such as lifting and bending [17,18]. The short form workstyle is a well-known instrument for evaluating the behavioral responses of workers in response to combined physical and psychosocial stressors at work [16,19,20,21,22,23]. These two instruments have been used in research studies about the problems of work-related musculoskeletal disorders in nurses, surgeons, office workers, and bus drivers in the past. [16,17,18,19,20,21,22,23,24,25] The main aim of this study is to compare the self-perceived physical demand of work and workstyle outcome of EM intervention versus conventional physiotherapy control (CO) intervention on individual workers with WRNSP and specific work demands. We hypothesized that this EM approach would contribute to a significant immediate and/or long-term improvement on the exposure of occupational related risk factors in people with WRNSP.

## 2. Materials and Methods 

### 2.1. Study Design

**Figure 1:**
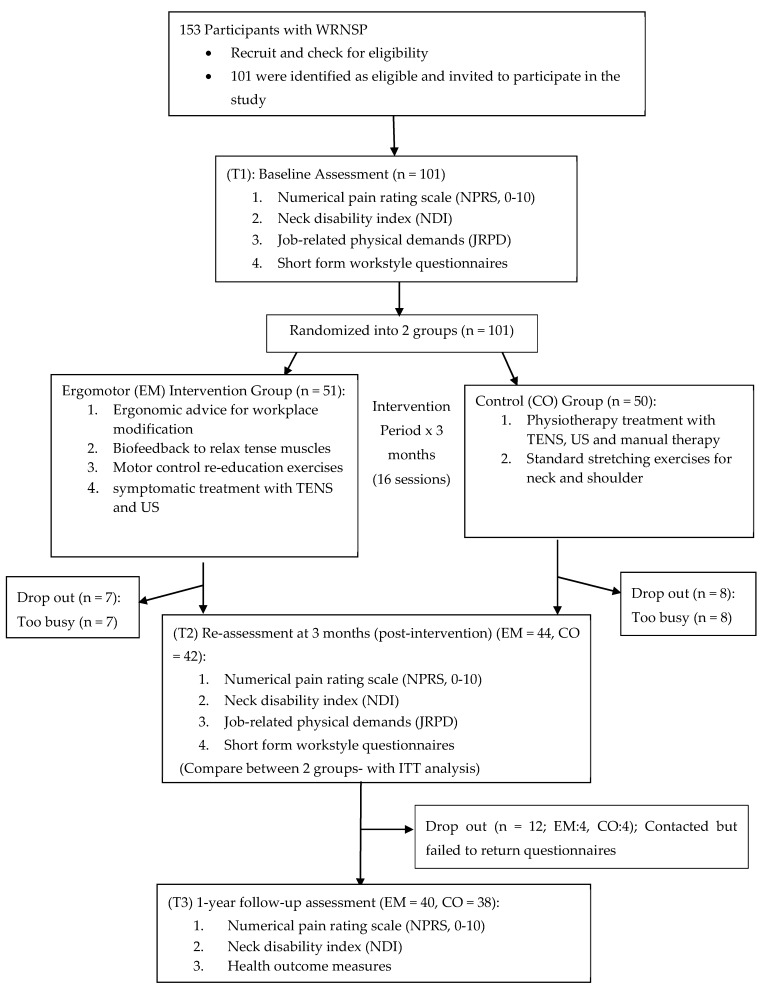
Flowchart of the overall study design.

A randomized controlled trial design was applied in this study. Selected participants were randomly assigned to either EM or CO interventions. Figure 1 illustrated the flow of this study with reference to the CONSORT guidelines [26].

### 2.2. Sample Size Planning

The “minimally clinically important difference” in pain score and function of 20% between-group differences was selected [27]. The corresponding Cohen’s d effect size is approximately 0.62. Given this effect size, a sample of 42 participants per group was required to achieve a power of 80% and a level of significance of 5% [28,29]. Assuming a 20% attrition rate, 50 participants per group were recruited in this study. Eventually, 51 participants met the inclusion criteria for the ergomotor group and participated fully in the whole study. 

### 2.3. Participants

One hundred and one individuals with WRNSP (51 males, 50 females) were recruited from two Physiotherapy Departments and two Department of Orthopedics and Traumatology outpatient clinics at two public hospitals in Hong Kong. Prior to the commencement of the study, human ethical approval was obtained from the local university (Reference number: HSEARS20141111002). 

All participants were recruited according to the inclusion criteria: (1) With full-time employment; (2) age range between 21 and 50; (3) met criteria for having WRNSP (average intensity during past four week of ≥2 on a 0–10 scale; (4) able to speak, read, and write in Cantonese. The study exclusion criteria were (1) neck pain caused by traumatic injury; (2) severe degenerative changes of the spine which were demonstrated on x-rays and/or MRI; (2) spinal stenosis with or without upper motor neuron lesion; (3) systematic diseases such as rheumatoid arthritis and/or ankylosing spondylitis; (4) specific diagnosis of neurological or musculoskeletal conditions of the upper extremity as the source of the referred symptoms of the neck pain; (3) fracture on neck or shoulder region. The inclusion and exclusion criteria of this study were based on previous research [28,29]. 

The randomization process was performed by a trained research assistant. Computer-generated random sequence table was used for the randomization. The research personnel responsible for data collection of the job-related physical demands and workstyle outcomes was blinded to the group assignment. 

### 2.4. Intervention Protocols

The EM intervention program was a 12-week program consisting of 16 sessions (60 min per session). The program was designed based on the finding from the baseline assessment, which included a detailed job task analysis. The program consisted of the following major components:

1. Ergonomics Knowledge Transfer Consultation (Ergo-): The therapists with ergonomics training, based on the findings from the comprehensive job demand analysis, advised the participant on the proper workplace adjustment. The ergonomics risk factors including working postures were explained to the participant. Suggestions on work equipment modification and task organization were given to participant if needed. The therapists mainly facilitated the participant to develop some workable low-tech ergonomics solutions to minimize the occupational risk factors. 

2. Biofeedback Motor Control Facilitation (Motor): The therapists with motor control training, based on the outcomes from the posture and biomechanical analysis, educated the participant on the optimum muscle control of the key neck and shoulder postural muscles (upper trapezius (UT) and lower trapezius (LT) muscles). In particular, the maintenance of the good head and scapula position, is an important emphasis in the exercises. This usually involves performing a “retraction” of the head (chin) posture as well as retraction of the scapula. Each participant was instructed to perform active exercises with the postural correction elements. The training protocol was based on our previous experience in training the UT activity in office workers [30]. In the once-a-week training session, the participant was also trained with wireless real-time surface EMG electrodes attached to the UT and LT muscles during a series of simulation activities. Virtual reality games were also used to train the participant to do some upper limb functional tasks. The participant could see the UT and LT muscle activities on an adjacent computer screen and received the visual feedback of the muscle activity. In addition, a small portable biofeedback device was applied to the participant. Once the participant learned the proper application of the biofeedback device, the device was loaned to the participant for both workplace and home training on a daily basis. 

3. Tailor-made neck and shoulder conditioning exercises: Specific conditioning exercises with prescription were taught to the participant to practice at home and at the workplace in order to reinforce the proper motor control. A logbook was provided to the participant to monitor the compliance of exercise training.

Participants in the CO group received 12-week conventional physiotherapy treatment including electrophysical modalities (ultrasound and transcutaneous electro nerve stimulation (TENS)) for symptomatic relief. The methods of application and dosage of each modality was standardized as much as possible and recorded. 

### 2.5. Occupational Exposure Measures

A detailed analysis of the job or functional tasks that are performed frequently by the participant contributing to the aggravation of symptoms was conducted. The Chinese version of the job-related physical demands (JRPD) form [31] and the workstyle short (WS) form [32] were used to assess the physical and psychosocial job demands of the participants at the (T1) pre- and (T2) post-intervention stages. The Chinese version of WS form was a validated tool to measure eight different psychosocial domains (1) pain; (2) social reactivity; (3) limited workplace support; (4) deadlines; (5) self-impose workplace; (6) break; (7) mood; and (8) autonomic response. Cheng and his colleagues reported that the Chinese version of WS had a high internal consistency (α = 0.84) and a good test-retest (3 weeks) reliability (r = 0.79 to 0.91). [22] The suggested cut-off point score of Workstyle short form is 28. The total point score ≥ 28 means “adverse” workstyle, for the total point score range from 0 to 27 which is considered as “healthy” workstyle. [20,21] These two instruments have been validated and commonly used to evaluate the physical and psychosocial risk factors in work-related musculoskeletal disorders in both research and clinical practice [33].

Other measurements included the self-reported pain score (0–10) and functional outcomes such as the neck disability index (NDI) and the disabilities of the arm, shoulder, and hand (DASH) questionnaire. These results have been reported in another publication [30]. We also evaluated the biomechanical measures such as surface electromyography and kinematic parameters of neck and shoulder performance [31].

In the 1-year follow-up (T3), the participants were contacted by phone and asked to rate their pain and neck function. They were also asked whether they had taken any sick leave and sought more treatment from medical doctor, physiotherapist, or TCM doctor.

### 2.6. Data Analysis

The outcome variables were compared between pre- (T1) and post-intervention (T2) assessment within each participant. The dropout cases were included according to the intention-to-treat method. Missing data were addressed by last value carried forward imputation method. The data were compared again with the post-intervention (T2) assessment results at 1-year follow-up (T3) for those outcomes that were examined at these two time points. Repeated measures ANOVA was performed for the dependent variables with the within-subject factors of trial (× 3 levels, T1,T2,T3) and between-subject factor of group (× 2 levels, intervention vs control). All statistical analyses were performed using SPSS (V.23.0, IBM, Armonk, NY, USA) for windows using a significance level of *p* < 0.05 (2-tailed) for all analysis to balance the type I and II errors.

## 3. Results

A total of 153 potential cases were screened and 101 participants were recruited after screening the inclusion and exclusion criteria. No significant difference was found in the baseline demographic characteristics between two groups (*p* > 0.05) (Table 1). Ninety participants successfully completed the 12-week interventions (EM group, *n* = 44; CO group, *n* = 46) (Figure 1). At 1-year follow-up, 78 patients were successfully contacted and completed the final follow-up. The dropout rate of post-intervention at (T2) and 1-year follow-up at the (T3) were 10% and 12% respectively.

### 3.1. Demographic Data of Participants

Table 1 shows the demographic data and the job categories of the participants at baseline. It is apparent that the participants came from a large variety of occupations and they all suffered from the common problem of WRNSP. There was a good balance of male and female participants. There were no significant differences of age, gender, body weight, and height between two groups.

### 3.2. Results on Occupational Exposure Measures

At the (T2) post intervention assessment, we also evaluated their occupational outcome measures to examine the physical and psychosocial effects at the workplace. This consisted of the JRPD and the WS questionnaires. Both the JRPD total score and the WS total scores showed significant improvement over time for both groups (Table 2). The extent of reduction in both these scores were greater in the EM group compared to the CO group. The JRPD (RPE) was the rate of perceived exertion for the tasks that were performed more than 4 hours per day. This variable showed a highly significant reduction in the (T2) post-intervention assessment in the EM group compared to the CO group. 

**Figure 2:**
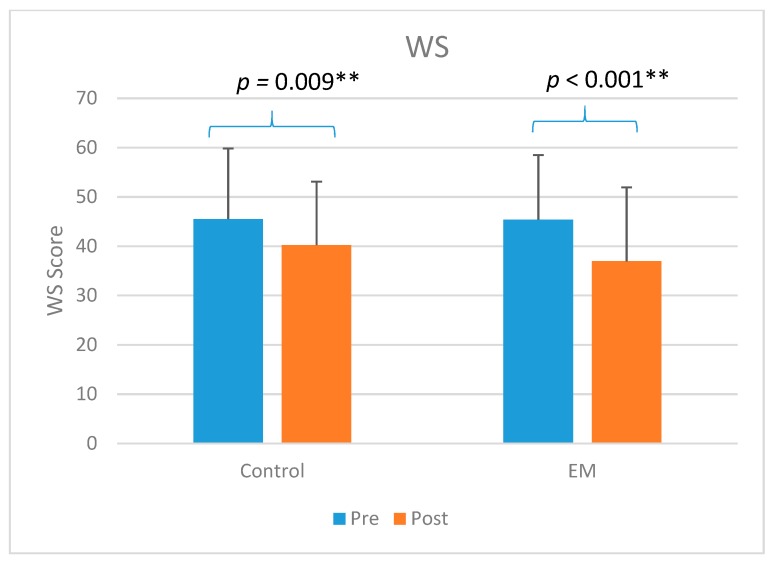
The comparison of the workstyle (WS) total score of control (CO) and (ergomotor) EM. ** significant at *p* < 0.01; Pre: pre-intervention; Post: post-intervention.

**Figure 3:**
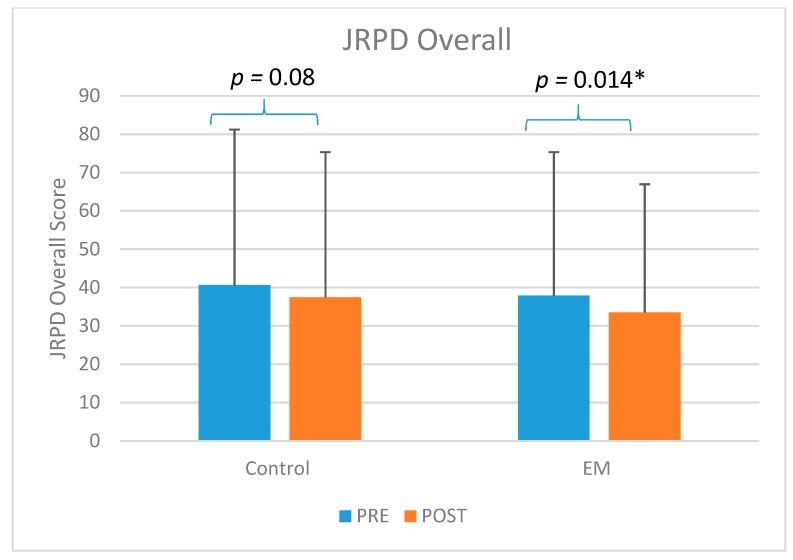
The comparison of the overall score of job-related physical demand (JRPD) of control (CO) and (ergomotor) EM. * significant at *p* < 0.05; Pre: pre-intervention; Post: post-intervention.

**Figure 4:**
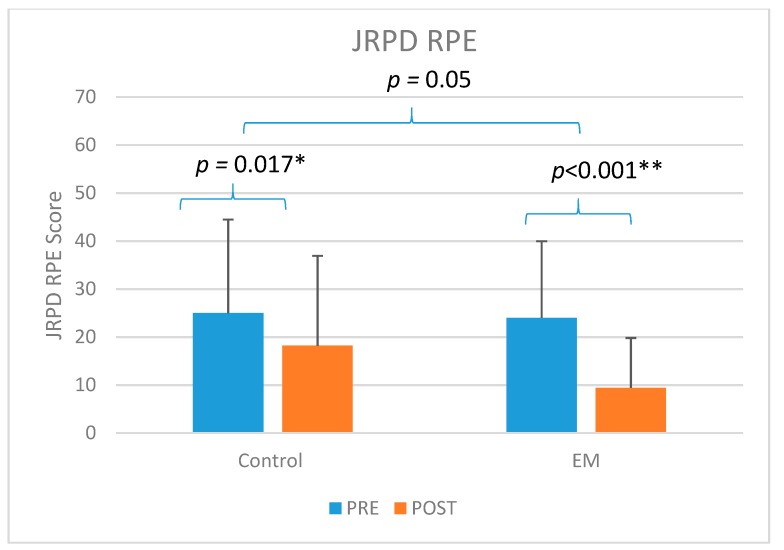
The comparison of the RPE score of job-related physical demand (JRPD) of control (CO) and (ergomotor) EM. * significant at *p* < 0.05, ** significant at *p* < 0.01.

Both EM (*p* < 0.001) and CO groups (*p* = 0.009) showed significant improvement in workstyle total score when comparing (T1) to (T2) (Figure 2). Only EM group (*p* = 0.014) showed significant improvement in the JRPD total score (*p* = 0.014) when comparing (T1) to (T2) (Figure 3). For the JRPD RPE Score, both EM group (*p* < 0.001) and CO group (*p* = 0.017) showed significant improvement (Figure 4).

The mean values for the number of visits and the total fees appeared to be lower for the EM group compared to the CO group. However, there was no statistically significant difference in these values comparing the two groups in the total healthcare costs using independent t-tests (t = 0.288, *p* = 0.774) (Table 3). 

## 4. Discussion

Both groups of participants reported significant improvement in pain scores and functional outcomes (NDI) with no significant difference between groups [30]. However, there were more apparent differences between groups when examining the occupational exposure measures such as the JRPD and workstyle. 

### 4.1. Effects of Ergomotor Approach on Job-Related Physical Demands

This study found significant improvement in both JRPD and WS in the participants who received the ergomotor intervention. The JRPD (job-related physical demands) questionnaire asked the participants to rate their working postures and movements in terms of “how often” they were exposed to these factors at their workplaces. We also asked the participants to report the perceived rate of exertion (RPE) for the five most frequently performed work tasks. In this part, the EM group showed a statistically significantly reduction at post-intervention from 24.0 ± 18.7 to 9.4 ± 10.4 compared to the control group (Pre: 25.0 ± 19.5; Post: 18.2 ± 16.0). In past research using the JRPD and the Borg scale to study the correlation between the low back musculoskeletal symptoms and the perceived exertion, a high level of internal consistency was reported between JRPD total score and perceived exertion [17]. The significant reduction in rating of perceived exertion in the present study may potentially be related to the changes made in the work practice or working postures by the participants in the EM Group. This finding may be one of the most important results from the present study, as it may be considered an indicator of the changes in the participants’ motor control habits resulting from the ergomotor training. As such, it may be an even more important outcome compared to the self-reported pain scores. The present results are also consistent with the findings of improved biomechanical measures of reduced muscle activity in the postural muscles such as UT, as reported in another paper [31]. However, it was difficult to provide precise data or standardized measurement on the different types of workplace modifications for the different job types. Future research should also consider the contemporary workplace environment with rapid advances in technology. 

The instrument of JRPD was used in earlier research studies in the United States on evaluating the physical work demands among office workers [18] and other job types within the military service [17]. The questions are phrased in a user-friendly manner, and able to capture the important occupational risk factors such as repetitive hand actions, static posture, frequency of bending, twisting of the trunk that are applicable to both sedentary and manual type of workers. While there are many other questionnaires that have been reported in the literature, the JRPD was considered the most appropriate one for the current sample of participants which consisted of a high proportion of office workers, as well as some participants with retail or manual work nature. Other commonly used instruments such as the job content questionnaire [33] was more focused on the evaluation of the psychosocial risk factors. The quick exposure checklist [34] and the rapid entire body assessment (REBA) [35] were commonly used observational methods to evaluate the physical demands on different job types in the different body regions such as the neck, back, upper and lower limbs. Takala et al. [36] has reviewed all the commonly used observational assessments for ergonomic analysis and provided a useful summary of the different methods. Future studies can consider using more than one method to evaluate the physical work demands in order to have more in-depth and precise data analysis on this topic. Biomechanical measurements such as spinal kinematics and electromyography may also provide more objective and real-time data on the physical loading in the workers performing different work tasks [37].

### 4.2. Effects of Ergomotor Approach on Workstyle 

There is a growing body of research indicating that there are relationship between an adverse workstyle and work-related musculoskeletal disorders of individuals in both developed and developing countries [7,20,22,23,38]. These studies have generally reported a high correlation of the workstyle total score or their sub-scales on different major factors such as “working through pain” and “social reactivity at work” to the work-related musculoskeletal symptom scores [22,23,32,38]. The workstyle (WS) questionnaire reflects the psychosocial components in the occupational exposure, and this comparison also showed more favorable results for the EM group (reduced from a mean of 45.4 to 36.9) compared to the control group (reduced from 45.5 to 40.2). This suggests that the ergomotor intervention training may have influenced both the physical and psychosocial aspects of the job exposure for the participants. The results of this study echoes with previous research that workplace-based exercise program and ergonomic intervention could improve occupational health of workers [20,21]. In this study, the ergomotor approach facilitated the workers to identify the adverse workstyles associated with WRNSP, which enhanced the awareness of the workplace ergonomic risk factors. 

### 4.3. Integrating Ergonomics and Motor Control Interventions

The current results have confirmed the importance of integrating ergonomic interventions into the clinical management of these patients and the training program needs to be transformed into actual workplace strategies [39]. Physiotherapists in the clinical setting as well as in the workplace setting should adopt this approach, and it will produce more long-term benefits for this group of patients [40]. When starting a course of treatment for such patients, physiotherapists should assess the work demands and ergonomic risks to musculoskeletal disorders of the patient in more details and design the exercise-training program and provide ergonomic advice to match the job demand. 

Nowadays, the rapid advances in technology enable the remote communication between therapists and patients, so that the advice on workplace adaptations do not need to take place in face-to-face consultations. There is also an abundance of information on the internet that is accessible to people, but the individual postural habits still require the advice from experienced clinicians. Our current approach combines the knowledge of ergonomics and the clinical expertise of physiotherapists focusing on how to integrate this knowledge into real-life applications during the actual daily work process. The results in this study showed positive effect on the occupational exposure outcomes, which aligns with a previous study on workplace ergonomics education combined with workplace-based exercise training to office workers [13,41,42]. The improvement in both subjective job related physical demand and workstyle may be an indicator of the positive knowledge and successful behavioral changes among the workers [42]. 

When therapists conduct motor control re-education for the patients, they should also try to design these exercises to simulate the work tasks of the patient. It is important that the patient can truly understand “how” their muscles are being activated when they perform different “work task” movements, and this is the benefit of incorporating EMG biofeedback training. This approach is the key factor that may produce long-term benefits for the patients. As these EMG devices are becoming more accessible and more reasonably priced, it is suggested that hospitals and clinics should consider incorporating these biomechanical measurement systems into their routine assessment of patients in the future.

### 4.4. Cost-Effectiveness of Ergomotor Approach 

In the 1-year follow-up, the participants were contacted by phone and asked to rate their pain and neck function. They were also asked whether they had taken any sick leave and sought more treatment from medical doctor, physiotherapist or TCM doctor. The results were difficult to interpret, with the CO group reporting more sessions of TCM treatment received whereas the EM group sought more treatment from private physiotherapists. However, the total number of sessions of all forms of treatment was higher in the CO group and total costs were also higher in this group. No firm conclusion can be drawn on the cost-effectiveness of the EM intervention program based on this result. Nonetheless, it has been suggested in the literature that multi-component interventions are warranted to manage occupational health problems and it is important to examine the “health economics” of these interventions [40]. 

### 4.5. Limitations of Study and Suggestions for Future Research

The present study involved a rather intensive intervention program, which requires a large amount of therapist time, and therefore we need to employ more than one therapist to conduct the EM and the CO interventions. Although we tried to brief all the therapists involved at the start of the study, there may be variations in individual management approach to treat the patients, and this may affect the outcomes of the study. 

It was not easy to control the dropout rates especially at the one-year follow-up. Ideally, it should be less than 20% from the baseline sample size. The final count of participants at the one-year follow-up was 78, which reflected a 22.8% dropout rate. In future study, a larger sample size at the start of the study may compensate for this problem and the small sample size may have contributed to the failure to reach statistical significance for some of the data. 

The program also has limitations in terms of the intervention design. While attempts were made to try simulating the different workplace situations of different participants, it is not always possible to achieve this because of the different environmental and work organization factors. For example, there was one participant who was a cook in a restaurant. We tried to adopt the exercises to involve the neck and shoulder regions in simulated actions of cooking, but it was difficult to find solutions to change the workstation arrangement. For those who were office workers, it was much easier to make suggestions for ergonomic changes to their workplaces, but this is not so easy for other workers such as drivers and the cook. In adopting the functional movements as outcome evaluations, these may also have limited generalizability, as they may not be matched to the physical job demands of different types of workers. 

The present study attempted to develop an “integrated” intervention approach that involved using different kinds of methods such as adding EMG biofeedback and attempting to simulate workplace tasks in the laboratory. There may be too much heterogeneity in the methods used and these cannot be standardized into clear-cut routines and dosages, this causes limitation in making the approach to have greater generalizability and better applicability for other clinicians to adopt this approach. The research team needs to work on developing better methods to standardize these intervention methods while still maintaining the individual tailoring of the program. 

## 5. Conclusions

The present study has demonstrated the effectiveness of the innovated ergomotor intervention program for workers with WRNSP to reduce their self-perceived physical demand and improve psychosocial health. The 12-week program consisted of ergonomics knowledge transfer consultation, biofeedback motor control facilitation, and tailor-made neck and shoulder conditioning exercises. The significant improvement in JRPD and WS were found at post-intervention, and this confirms the the effectiveness of the program on the occupational risk factors. There are potential applications of this intervention model for workers with other work-related musculoskeletal disorders including lower back pain and lower limb disorders. 

## Figures and Tables

**Table 1:** Baseline demographic characteristics of the control (CO) group (*n* = 50) and ergomotor (EM) group (*n* = 51).

		CO Group		EM Group		*p*
Age (yr)		36.4 ± 8.9	(22–54)	35.6 ± 8.7	(20–49)	0.494
Gender	Male	26	52%	25	49%	0.766
	Female	24	48%	26	51%	
Weight (kg)		64.4 ± 13.7	(39.1–101.2)	62.6± 11.8	(42.3–82.7)	0.561
Height (cm)		167.9 ± 10.2	(145.0–188.5)	166.4 ± 9.3	(145.0–180.0)	0.734
BMI (kg/m^2^)		22.7± 3.8	(16.6–32.3)	22.4± 2.6	(17.6–29.3)	0.529
Job categories						
	Banking and Finance	2	4.0%	6	11.76%	
	Food and Catering			1	1.96%	
	Engineering	4	8.0%	1	1.96%	
	Photographer/Tourism	2	4.0%	1	1.96%	
	Education (primary, secondary school teacher)	2	4.0%	4	7.84%	
	Healthcare	7	14.0%	6	11.76%	
	Sales and Retail	5	10.0%			
	Clerical/Admin	15	30.0%	19	37.25%	
	IT	3	6.0%	2	3.92%	
	Driver	2	4.0%	1	1.96%	
	Academic	8	16.0%	10	19.61%	
	Total	50	100.0%	51	100.00%	

NOTE: values are mean (range) or *n* (%). Abbreviations: BMI, body mass index. Age, weight, height, BMI are shown as mean (range), gender is presented as count (%).

**Table 2:** Summary of occupational exposure measures at pre- and post-intervention.

Occupational Exposure Measure	(T1) Pre-Intervention		(T2) Post-Intervention		Statistical Analysis (Univariate) F, *p* Values		
	EM	CO	EM	CO	Time	Group	Time * Group
JRPD (total)	37.8(14)	40.6(14.5)	33.5(13.5)	37.5(12.5)	F_1,99_ = 9.05, *p* = 0.003 **	F_1,99_ = 1.94, *p* = 0.166	F_1,99_ = 0.24, *p* = 0.623
JRPD (RPE)	24.0(18.7)	25.0(19.5)	9.4(10.4)	18.2(16)	F_1,99_ = 29.61, *p* < 0.000 **	F_1,99_ = 3.43, *p* = 0.067	F_1,99_ = 3.93, *p* = 0.05 *
WS (total)	45.4(13.1)	45.5(14.3)	36.9(15)	40.2(12.9)	F_1,99_ = 24.26, *p* < 0.000 **	F_1,99_ = 0.51, *p* = 0.476	F_1,99_ = 1.28, *p* = 0.260

* significant at *p* < 0.05, ** significant at *p* < 0.01.

**Table 3:** Summary of health outcome measures at one-year follow-up.

	NUMBER OF SESSIONS		FEES PER SESSION (Estimated in HKD)	TOTAL FEES (HKD)	
	CO Group	EM Group		CO Group	EM Group
Med Doctor_public	6	0	100	600	0
Med Doctor_private	10	2	300	3000	600
Physiotherapy_public	8	18	60	480	1080
Physiotherapy_private	31	53	500	15,500	26,500
TCM_public	0	1	100	0	100
TCM_private	147	88	300	44,100	26,400
Total sessions	202	162	Total fees	63,680	54,680

Med: Medical; TCM: Traditional Chinese Medicine.

## References

[1] Buckle P.W., Devereux J.J. (2002). The nature of work-related neck and upper limb musculoskeletal disorders. Appl. Ergon..

[2] Chen X., O’Leary S., Johnston V. (2018). Modifiable individual and work-related factors associated with neck pain in 740 office workers: A cross-sectional study. Braz. J. Phys. Ther..

[3] Shum S.-L. (2008). Occupational Risk Factors for Neck and Shoulder Pain Among Hong Kong Nurses, S.

[4] Van Eerd D., Munhall C., Irvin E., Rempel D., Brewer S., Van Der Beek A.J. (2016). Effectiveness of workplace interventions in the prevention of upper extremity musculoskeletal disorders and symptoms: An update of the evidence. Occup. Environ. Med..

[5] Punnett L., Wegman D.H. (2004). Work-related musculoskeletal disorders: The epidemiologic evidence and the debate. J. Electromyogr. Kinesiol..

[6] Kwok H.K.H., Szeto G.P.Y., Cheng A.S.K., Siu H., Chan C.C.H. (2011). Occupational rehabilitation in Hong Kong: Current status and future needs. J. Occup. Rehabil..

[7] Cheung K., Szeto G., Lai G., Ching S. (2018). Prevalence of and Factors Associated with Work-Related Musculoskeletal Symptoms in Nursing Assistants Working in Nursing Homes. Int. J. Environ. Res. Public Health.

[8] Umer W., Antwi-Afari M.F., Li H., Szeto G.P., Wong A.Y. (2018). The prevalence of musculoskeletal symptoms in the construction industry: A systematic review and meta-analysis. Int. Arch. Occup. Environ. Health.

[9] Xu Y.W., Cheng A.S., Li-Tsang C.W. (2013). Prevalence and risk factors of work-related musculoskeletal disorders in the catering industry: A systematic review. Work.

[10] Verhagen A.P., Karels C., Bierma-Zeinstra S.M., Feleus A., Dahaghin S., Burdorf A., Koes B.W. (2007). Exercise proves effective in a systematic review of work-related complaints of the arm, neck, or shoulder. J. Clin. Epidemiol..

[11] Larsson B., Søgaard K., Rosendal L. (2007). Work related neck-shoulder pain: A review on magnitude, risk factors, biochemical characteristics, clinical picture and preventive interventions. Best Pract. Res. Clin. Rheumatol..

[12] Dainoff M.J., Aarås A., Horgen G., Konarska M., Larsen S., Thoresen M., Cohen B.G. (2005). The effect of an ergonomic intervention on musculoskeletal, psychosocial and visual strain of VDT entry work: organization and methodology of the international study. Int. J. Occup. Saf. Ergon..

[13] Ting J.Z.R., Chen X., Johnston V. (2019). Workplace-Based Exercise Intervention Improves Work Ability in Office Workers: A Cluster Randomised Controlled Trial. Int. J. Environ. Res. Public Health.

[14] Ma C., Szeto G.P., Yan T., Wu S., Lin C., Li L. (2011). Comparing biofeedback with active exercise and passive treatment for the management of work-related neck and shoulder pain: A randomized controlled trial. Arch. Phys. Med. Rehabil..

[15] Johnston V., O’Leary S., Comans T., Straker L., Melloh M., Khan A., Sjøgaard G. (2014). A workplace exercise versus health promotion intervention to prevent and reduce the economic and personal burden of non-specific neck pain in office personnel: Protocol of a cluster-randomised controlled trial. J. Physiother..

[16] So B., Cheng A., Szeto G. (2017). Cumulative IT Use Is Associated with Psychosocial Stress Factors and Musculoskeletal Symptoms. Int. J. Environ. Res. Public Health.

[17] Daniels C., Huang G.D., Feuerstein M., Lopez M. (2005). Self-report measure of low back-related biomechanical exposures: Clinical validation. J. Occup. Rehabil..

[18] Dane D., Feuerstein M., Huang G.D., Dimberg L., Ali D., Lincoln A. (2002). Measurement properties of a self-report index of ergonomic exposures for use in an office work environment. J. Occup. Environ. Med..

[19] Feuerstein M., Nicholas R.A., Huang G.D., Dimberg L., Ali D., Rogers H. (2004). Job stress management and ergonomic intervention for work-related upper extremity symptoms. Appl. Ergon..

[20] Meijer E.M., Sluiter J.K., Frings-Dresen M.H. (2008). Is workstyle a mediating factor for pain in the upper extremity over time?. J. Occup. Rehabil..

[21] Harrington C.B., Siddiqui A., Feuerstein M. (2009). Workstyle as a predictor of pain and restricted work associated with upper extremity disorders: A prospective study. J. Hand Surg. Am..

[22] Cheng A.S., Szeto G.P., Xu Y.W., Feuerstein M. (2014). Chinese translation and cross cultural adaptation of the workstyle short form. J. Occup. Rehabil..

[23] Cheung K., Ching S., Ma K., Szeto G. (2018). Psychometric Evaluation of the Workstyle Short Form among Nursing Assistants with Work-Related Musculoskeletal Symptoms. Int. J. Environ. Res. Public Health.

[24] Szeto G.P., Lam P. (2007). Work-related musculoskeletal disorders in urban bus drivers of Hong Kong. J. Occup. Rehabil..

[25] Szeto G.P., Ho P., Ting A.C., Poon J.T., Cheng S.W., Tsang R.C. (2009). Work-related musculoskeletal symptoms in surgeons. J. Occup. Rehabil..

[26] Schulz K.F., Altman D.G., Moher D. (2010). CONSORT 2010 Statement: Updated guidelines for reporting parallel group randomised trials. J. Clin. Epidemiol..

[27] Ferreira M.L., Herbert R.D., Ferreira P.H., Latimer J., Ostelo R.W., Nascimento D.P., Smeets R.J. (2012). A critical review of methods used to determine the smallest worthwhile effect of interventions for low back pain. J. Clin. Epidemiol..

[28] Maher C.G. (2013). Natural course of acute neck and low back pain in the general population: The HUNT study. Pain.

[29] Cohen S.P. (2015). Epidemiology, diagnosis, and treatment of neck pain. Mayo Clin. Proc..

[30] Tsang S.M., So B.C., Lau R.W., Dai J., Szeto G.P. (2019). Comparing the effectiveness of integrating ergonomics and motor control to conventional treatment for pain and functional recovery of work-related neck-shoulder pain: A randomized trial. Eur. J. Pain.

[31] Tsang S.M., So B.C., Lau R.W., Dai J., Szeto G.P. (2018). Effects of combining ergonomic interventions and motor control exercises on muscle activity and kinematics in people with work-related neck-shoulder pain. Eur. J. Appl. Physiol..

[32] Nicholas R.A., Feuerstein M., Suchday S. (2005). Workstyle and upper-extremity symptoms: A biobehavioral perspective. J. Occup. Environ. Med..

[33] Santos K.O.B., de Araújo T.M., Carvalho F.M., Karasek R. (2017). The job content questionnaire in various occupational contexts: Applying a latent class model. BMJ Open.

[34] Oliv S., Gustafsson E., Baloch A.N., Hagberg M., Sandén H. (2019). The Quick Exposure Check (QEC)—Inter-rater reliability in total score and individual items. Appl. Ergon..

[35] McAtamney H.S., Stanton N., Hedge A., Salas E., Hendrick H.W. (2005). Rapid Entire Body Assessment. Handbook of Human Factors and Ergonomics Methods.

[36] Takala E.P., Pehkonen I., Forsman M., Hansson G.Å., Mathiassen S.E., Neumann W.P. (2010). Systematic evaluation of observational methods assessing biomechanical exposures at work. Scand. J. Work Environ. Health.

[37] Harari Y., Bechar A., Riemer R. (2019). Workers’ biomechanical loads and kinematics during multiple-task manual material handling. Appl. Ergon..

[38] Sharan D., Parijat P., Sasidharan A.P., Ranganathan R., Mohandoss M., Jose J. (2011). Workstyle risk factors for work related musculoskeletal symptoms among computer professionals in India. J. Occup. Rehabil..

[39] Hoe V.C., Urquhart D.M., Kelsall H.L., Zamri E.N., Sim M.R. (2018). Ergonomic interventions for preventing work-related musculoskeletal disorders of the upper limb and neck among office workers. Cochrane Database Syst. Rev..

[40] Prall J., Ross M. (2019). The management of work-related musculoskeletal injuries in an occupational health setting: The role of the physical therapist. J. Exerc. Rehabil..

[41] Pereira M., Comans T., Sjøgaard G., Straker L., Melloh M., O’leary S. (2019). The impact of workplace ergonomics and neck-specific exercise versus ergonomics and health promotion interventions on office worker productivity: A cluster-randomized trial. Scand. J. Work Environ. Health.

[42] Mani K., Provident I., Eckel E. (2016). Evidence-based ergonomics education: Promoting risk factor awareness among office computer workers. Work.

